# Supplementary material for: Text message reminders for improving sun protection habits: A systematic review
Source: PLoS One. 2020 May 19;15(5):e0233220. doi: 10.1371/journal.pone.0233220 (PMC7236986; doi:10.1371/journal.pone.0233220)
Supplement: S1 Material — (DOCX) [file pone.0233220.s002.docx]

## Supplementary material 1: search strategy

| **Database** | **Search term** | **Date** | **Results** |
| --- | --- | --- | --- |
| Pubmed | (((("text messaging"[MeSH Terms] OR text*[Title/Abstract] OR "message service"[Title/Abstract] OR SMS[Title/Abstract] OR txt[Title/Abstract] OR reminder system*[Title/Abstract] OR messag*[Title/Abstract] OR remind*[Title/Abstract] OR "messaging system"[Title/Abstract]) OR ("telemedicine"[MeSH Terms] OR telehealth[Title/Abstract] OR telemonitor[Title/Abstract] OR mHealth[Title/Abstract] OR eHealth[Title/Abstract] OR "mobile health"[Title/Abstract]) OR ("cell phone"[MeSH Terms] OR "mobile telephone"[Title/Abstract] OR "mobile phone"[Title/Abstract] OR cellular phon*[Title/Abstract] OR "smartphone"[MeSH Terms]) OR ("wireless technology"[MeSH Terms] OR "wearable technology"[Title/Abstract]) OR “mobile”[Title/Abstract]))) AND (("sunscreening agents"[MeSH Terms] OR sunscreen*[Title/Abstract] OR "lip balm"[Title/Abstract]) OR ("sunburn"[MeSH Terms] OR sunburn*[Title/Abstract]) OR ("protective clothing"[MeSH Terms] OR "sun protection"[Title/Abstract] OR "eye protective devices"[MeSH Terms] OR goggles[Title/Abstract] OR sunglasses[Title/Abstract] OR "safety lenses"[Title/Abstract] OR "safety glasses"[Title/Abstract] OR "eyeglasses"[MeSH Terms]) OR ("sunlight"[MeSH Terms] OR sunshine[Title/Abstract] OR "ultraviolet rays"[MeSH Terms] OR ultra-violet ray*[Title/Abstract] OR UV light[Title/Abstract] OR actinic ray*[Title/Abstract] OR "UV radiation"[Title/Abstract]) OR (shade[Title/Abstract] OR shadow[Title/Abstract]) OR (midday[Title/Abstract]) OR (outdoors[Title/Abstract]) OR skin cancer[Title/Abstract] OR photo-protection[Title/Abstract] OR sun-protection[Title/Abstract] OR sun safety[Title/Abstract] OR melanoma[Title/Abstract] OR “skin self-examination” [Title/Abstract] OR “indoor tanning” [Title/Abstract])) AND ((clinical[Title/Abstract] AND trial[Title/Abstract]) OR clinical trials as topic[MeSH Terms] OR clinical trial[Publication Type] OR random*[Title/Abstract] OR random allocation[MeSH Terms] OR therapeutic use[MeSH Subheading]) | December 2018 | 784 |
| Scopus | (INDEXTERMS(("text messaging" OR telemedicine OR "cell phone" OR smartphone OR "wireless technology") AND ("sunscreening agents" OR sunburn OR "protective clothing" OR "eye protective devices" OR eyeglasses OR sunlight OR "ultraviolet rays")) OR (TITLE-ABS-KEY (("text messaging" OR text* OR "message service" OR sms OR txt OR "reminder system" OR messag* OR remind* OR "messaging system" OR telemedicine OR telehealth OR telemonitor OR mHealth OR eHealth OR cellphone OR smartphone OR "wireless technology" OR "wearable technology" OR mobile) AND ("sunscreening agents" OR sunscreen* OR "lip balm" OR sunburn* OR "protective clothing" OR "sun protection" OR "eye protective devices" goggles OR sunglasses OR "safety lenses" OR "safety glasses" OR eyeglasses OR sunlight OR sunshine OR "ultraviolet rays" OR "UV light" OR "actinic rays" OR "UV radiation" OR shade OR shadow OR midday OR outdoors OR "skin cancer" OR photoprotection OR "sun safety" OR melanoma OR “skin self-examination” OR "indoor tanning")))) AND (INDEXTERMS ("clinical trials" OR "clinical trials as a topic" OR "randomized controlled trial" OR "Randomized Controlled Trials as Topic" OR "controlled clinical trial" OR "Controlled Clinical Trials" OR "random allocation" OR "Double-Blind Method" OR "Single-Blind Method" OR "Cross-Over Studies" OR "Placebos" OR "multicenter study" OR "double blind procedure" OR "single blind procedure" OR "crossover procedure" OR "clinical trial" OR "controlled study" OR "randomization" OR "placebo")) OR (TITLE-ABS-KEY (("clinical trials" OR "clinical trials as a topic" OR "randomized controlled trial" OR "Randomized Controlled Trials as Topic" OR "controlled clinical trial" OR "Controlled Clinical Trials as Topic" OR "random allocation" OR "randomly allocated" OR "allocated randomly" OR "Double-Blind Method" OR "Single-Blind Method" OR "Cross-Over Studies" OR "Placebos" OR "cross-over trial" OR "single blind" OR "double blind" OR "factorial design" OR "factorial trial"))) OR (TITLE-ABS (clinical trial* OR trial* OR rct* OR random* OR blind*)) | December 2018 | 134 |
| CENTRAL | 1. MeSH descriptor: [Text Messaging] this term only  2. MeSH descriptor: [Telemedicine] this term only  3. MeSH descriptor: [Cell Phone] this term only  4. MeSH descriptor: [Smartphone] this term only  5. MeSH descriptor: [Wireless technology] this term only  6. text*:ti,ab or message service:ti,ab or SMS:ti,ab or txt:ti,ab or reminder system*:ti,ab or messag*:ti,ab or remind*:ti,ab or messaging system:ti,ab or telehealth:ti,ab or telemonitor:ti,ab or mHealth:ti,ab or eHealth:ti,ab or cellular phon*:ti,ab or wearable technology:ti,ab or mobile:ti,ab  7. MeSH descriptor: [Sunscreening Agents] this term only  8. MeSH descriptor: [Sunburn] this term only  9. MeSH descriptor: [Protective Clothing] this term only  10. MeSH descriptor: [Eye Protective Devices] this term only  11. MeSH descriptor: [Eyeglasses] this term only  12. MeSH descriptor: [Sunlight] this term only  13. MeSH descriptor: [Ultraviolet Rays] this term only  14. sunscreen*:ti,ab or lip balm:ti,ab or sunburn*:ti,ab or sun protection:ti,ab or goggles:ti,ab or sunglasses:ti,ab or safety lenses:ti,ab or safety glasses:ti,ab or sunshine:ti,ab or ultra-violet ray*:ti,ab or UV light:ti,ab or actinic ray*:ti,ab or UV radiation:ti,ab or shade:ti,ab or shadow:ti,ab or midday:ti,ab or outdoors:ti,ab or skin cancer:ti,ab or photo-protection:ti,ab or sun-protection:ti,ab or sun safety:ti,ab or melanoma:ti,ab or skin self-examination:ti,ab or indoor tanning:ti,ab  15. #1 or #2 or #3 or #4 or #5 or #6  16. #7 or #8 or #9 or #10 or #11 or #12 or #13 or #14  17. #15 and #16 (final search term) | December 2018 | 415 |
