# Supplementary material for: Text message reminders for improving sun protection habits: A systematic review
Source: PLoS One. 2020 May 19;15(5):e0233220. doi: 10.1371/journal.pone.0233220 (PMC7236986; doi:10.1371/journal.pone.0233220)
Supplement: S3 Material — (DOCX) [file pone.0233220.s004.docx]

## Supplementary material 3: study characteristics of individual studies, in detail.

| **First author, year (country)** | **Design** | **Population, age, and inclusion/exclusion criteria** | **Number of patients at baseline*** | **Text message intervention** | **Participants’ input** | **Sun protection outcomes** | **Follow-up** | **Outcomes effects** | **Funding** |
| --- | --- | --- | --- | --- | --- | --- | --- | --- | --- |
| Duffy, 2018 (United States) | Parallel RCT | Outdoor workers (operating engineers)  Age: ≥18 years of age. Mean age: 44.2 years  Inclusion: interest in enrolling in the sun protection study; owned a cell phone that accepted text messages; and willing to share their phone number with the research institution. | Education only: 91  Education and SMS reminders: 86  Education and mails: 87  Education, SMS, and mails: 93 | **Text message intervention**   1. **What:** A text bank of positives messages guided by the health belief model was first drafted by faculty and students, and then modified with feedback from a few Operating Engineers. 2. **When and how much:**  - Date: messages were delivered between summer and autumn of the northern hemisphere - Frequency: text messages were delivered in three random weekdays. - Hour: between 8 and 10 a.m. - Duration: five months. (May – September)  1. **Tailoring:** Use of the HBM profile to tailor the messages 2. **How well (planned):** Adherence or fidelity were not assessed.   **Other interventions**  **Education only:** 30-min PowerPoint presentation during their annual safety training. It included information on: the current use of sun protection among local members, incidence and prevalence of skin cancer, types of skin cancers and risk factors, and methods to prevent sun burning.  **Education and text message reminders:** in addition to the educational presentation, this group also received cell phone text messages.  **Education and mailed sunscreen:** in addition to the educational presentation, this group also received mailed sunscreen three times over the summer, including large bottles of SPF30 lotion and a small bottle that could be refilled and attached to their key rings.  **Education, mailed sunscreen, and text message reminders:** this group received the education presentation, text message reminders, and mailed sunscreen. | Authors tailored their messages according to the HBM profile of their participants. | **Get at least one sunburn:** yes or no  **Sunscreen use:** never, some of the time, about half the time, most of the time, always  **Number of sunburns:** 0, 1, 2, 3, 4 or more | 5 months | - **At least one sunburn: First comparison^1^:** (I: 50/77 = 64.9%) vs (C: 40/67=59.7%). **Second comparison ^1^**: (I: 42/69 = 60.9%) vs (C: 57/80= 71.3%) - **Sunscreen use (5-point Likert scale: from never to always): First comparison ^1^:** (I: n=77, mean 2.8 ± 1.0) vs (C: n=67, mean: 2.7 ± 1.2). **Second comparison ^1^**: (I: n=69, mean: 2.4 ± 1.1) vs (C: n=80, mean: 2.2 ± 1.2) - **Number of sunburns: First comparison ^1^:** (I: n=77, mean: 1.2 ± 1.2) vs (C: n=67, mean: 1.2 ± 1.2). **Second comparison ^1^**: (I: n=69, mean: 1.2 ± 1.3 arm) vs (C: n=80, mean: 1.4 ± 1.2 arm) | The Blue Cross Blue Shield of Michigan Foundation |
| Darlow, 2017 (United States) | Parallel RCT | Women from a metropolitan region at the northeast of the United States  Age: 18-29 years (M: 24.4)  Inclusion: moderate to high risk of skin cancer based on The Brief Skin Cancer Risk Assessment Tool and owned a mobile phone.  Exclusion: those with a personal history of skin cancer. | Assessment only: 24  Tailored text messages: 24  Behavior-tracking messages: 28  Tailored text messages and behavior tracking messages: 28  It is not clear which groups are being compared to get the results that are presented in the paper. | **Main intervention**  **Tailored Text Messaging:** participants were sent a tailored text message every morning.   1. **What:** Tailored messages were developed by the study investigators to apply to each of the health belief model constructs. A total of 22 messages were developed, with 3 to 6 messages applying to each HBM construct. 2. **When and how much**  - Date: dates of beginning and finishing are not described - Frequency: every day - Hour: 9:00 a.m. - Duration: two weeks  1. **Tailoring:** Tailored messages were delivered to participants according to personal scores for each HBM constructs, which was assessed at baseline. Examples of constructs are perceived benefits of sun protection, perceived barriers of sun protection, perceived susceptibility of skin cancer, self-effectiveness for sun protection ad cues to action for sun protection.   Authors used previous focus group where people criticized the messages’ content.   1. **How well (planned):** Adherence or fidelity were not assessed for this intervention.   **Other interventions**  **Assessment only:** participants were only assessed online using the Brief Skin Cancer Risk Assessment Tool, and internally consistent measure designed to identify those at phenotypic risk of developing skin cancer. Assessment only was three times (baseline, post assessment, and 4-week follow up [2 weeks after post assessment]).  **Behavior Tracking System:** it was performed for two weeks and began every day at 5:00 p.m. Entries began in the evening and first prompted participants to indicate if they tanned that day or not and why they tanned. Participants who indicated that they tanned that day received follow-up questions asking why they tanned and if they tanned indoors, outdoors, or both. Moreover, they were asked to indicate if they protected their skin from the sun during the day or not. If they answered no, they were asked to indicate why they did not protect their skin. If they said yes, they were asked to indicate how they protected their skin.  **Tailored text messaging and behavior tracking:** participants randomized to this group received both interventions (tailored text messages and behavior tracking system). | - Authors tailored their messages according to the HBM profile of their participants. - Authors developed the intervention messages using previous focus groups feedback, where participants criticized the content of messages. | **Ultraviolet radiation exposure behaviors** adapted by Ingledew, Ferguson, and Markland 2010: it was measured using an adapted scale which assesses the frequency (1 = never; 5 = always) of ultraviolet radiation exposure behaviors (wearing clothes that expose the skin to the sun, using sunlamps or tanning beds, sunbathing, and using products to get a faster or deeper tan) over the past month.  **Sun protection habits** adapted from Glanz 2008:   - **Sunscreen use**: it was an adapted item from other investigators. Participants indicated how often they engaged to sunscreen use (1 = never; 5 = always). The mean was calculated assessing two items (one for sunscreen on the face, the other for sunscreen on the rest of the body). - **Wearing a hat**: it was an adapted item from other investigators. Participants indicated how often they engaged to wearing a hat (1 = never; 5 = always). - **Staying in shade**: it was an adapted item from other investigators. Participants indicated how often they engaged in staying in shade (1 = never; 5 = always). | 4 weeks | In the Results section of the paper, authors stated that:   - **Ultraviolet radiation exposure behaviors (5-points likert scale):** those who received the SMS group reported a higher score, relative to those who did not (mean = 2.2 ± 0.6 vs 2.1 ± 0.5). - **Wearing a hat (5-points likert scale):** those who received SMS were more likely to wear a hat in the past month, relative to those who did not (mean = 2.0 ± 1.0 vs 1.8 ± 0.9).   The paper does not bring enough information in order to calculate MDs and their 95% confidence intervals. | The Aetna Foundation |
| Youl, 2015  &  Janda, 2013  (Australia) | Parallel RCT | Participants from the Queensland electoral and Medicare rolls  Age: 18 to 42 years of age  Inclusion: indicated interest by e-mail, text message, or returned mail, and completed the baseline telephone interview. | Sun protection messages: 187  Skin self-examination messages: 176  Physical activity messages: 183 | **Intervention**  The intervention group received weekly text messages reminders about sun protection. This routine ended in the first 12 weeks, then, participants were requested to complete an interview, after this, they received the messages monthly for the following nine months.   1. **What:** Messages aimed to address the constructs of the social cognitive model, such as increasing self-effectiveness, building behavioral capacity, or guiding outcome expectations. 2. **When and how much**  - Date: southern hemisphere winter of 2013 (intervention ended in August) - Frequency: weekly for the first 12 weeks and monthly the following nine months - Hour: not described - Duration: 12 months  1. **Tailoring:** Messages were personalized using participants’ name and gender, skin cancer risk factors, number of times being sunburnt, previous performance of skin self-examinations. This information was collected through a previous pilot survey. 2. **How well (planned):** Adherence or fidelity were not assessed.   **Control Skin self-examination messages.** This group received weekly text messages reminders about skin self-examination. This routine ended in the first 12 weeks, then, participants were requested to complete an interview, after this, they received the messages monthly for the following nine months.  **Physical activity messages.** This group received weekly text messages reminders about physical activity. This routine ended in the first 12 weeks, then, participants were requested to complete an interview, after this, they received the messages monthly for the following nine months. | Authors collected a pilot survey to estimate the participants’ preferences of the SMS text messages content. | **Sun protection score:** it averages the frequency (4-point Likert scale [1 = never/rarely, 4 = always]) of wearing a shirt with sleeves, wearing sunglasses, staying in the shade, using sunscreen, limiting time in the sun during midday, and wearing a hat.  **Skin self-examination:** yes or no, thoroughness (any, part-body, whole-body)  **Sunburn:** any, 2 or more  **Attempted suntan:** yes or no | 3 months  &  12 months | - **Sun protection habits index (ranging 1/never – 4/always):** (**FU: 3 months):** (I: n=181, mean: 2.54 ± 0.4) vs (C: n=164, mean: 2.52 ± 0.48). (**FU: 12 months):** (I: n=178, mean: 2.63 ± 0.46) vs (C: n=166, mean: 2.50 ± 0.50) - **Any skin self-examination (SSE) in past 3 months: (FU: 3 months)**: (I: 64/176 = 36.4%) vs (C: 53/164= 32.3%). **(FU: 12 months)**: (I: 83/173 = 48%) vs (C: 65/165= 39.2%) - **Whole-body SSE at time of last SSE: (FU: 3 months)**: (I: 35/176= 19.9%) vs (C: 32/164= 19.5%). **(FU: 12 months)**: (I: 24/173= 13.9%) vs (C: 18/165= 10.9%) - **Any sunburn in the past 12 months (FU: 12 months)**: (I: 121/173 = 69.9%) vs (C: 120/165=72.7%) - **Two or more sunburns in past 12 months (FU: 12 months)**: (I: 60/173= 34.7%) vs (C: 65/165= 39.4%) - **Attempted suntan in past 12 months (FU: 12 months)**: (I: 26/173= 15%) vs (C: 26/165= 15.8%) | Australian National Health and Medical Research Council (NHMRC) |
| Gold, 2011 (Australia) | Parallel RCT | Individuals residing in the state of Victoria, Australia  Age: 16 - 29 years of age  Inclusion: subscribed to a mobile advertising service  Exclusion: not described | I: 200; C: 158 | **Intervention**  The intervention group received eight text messages reminders fortnightly during Australia summer period. Messages aimed to increase knowledge, reinforce behaviors, change attitudes and increased perceived behavioral control about sun protection.   1. **What:** Initial messages were developed by the investigators and staff at the Burnet Institute, and focus tested with young people. It was based primarily on Weinstein’s Precaution Adoption Process model and incorporated elements from Ajzen’s Theory of Planned Behaviors and Bandura’s concept of self-effectiveness. Messages were humorous, short, used informal language and were linked to particular annual events where possible. 2. **When and how much**  - Date: messages were delivered between the southern hemisphere summer (2008) and autumn (2009). - Frequency: fortnightly over the summer period. - Hour: afternoon. - Duration: four months   **Tailoring:** Authors used previous focus group where people criticized the messages’ content.   1. **How well (planned):** Adherence or fidelity were not assessed.   **Control** The control group received eight text messages reminders fortnightly during the Australia summer period. Messages aimed to increase knowledge, reinforce behaviors, change attitudes and increased perceived behavioral control about safer sex protection. Messages were humorous, short, used informal language and were linked to particular annual events where possible. | Authors developed the intervention messages using previous focus groups feedback, where participants criticized the content of messages. | **Preference for a dark tan:** yes or no  **Hat-wearing frequency:** never/rarely/sometimes or usually/always  **Sunscreen wearing frequency:** never/rarely/sometimes or usually/always  **Frequency of seeking shade:** never/rarely/sometimes or usually/always  **Frequency of wearing deliberately skimpy clothing:** never/rarely/sometimes or usually/always | 4 months | - **Preference for a dark tan:** (I: 20/200 = 10%) vs (C: 14/158 = 8.86%) - **Believe about risk of cancer (consideration of the long-term consequences related to cancer risk):** (I: 116/200 = 58%) vs (C: 91/158 =57.6%) - **Usually/always wears hat:** (I: 63/200 = 31.5%) vs (C: 45/158 = 28.5%) - **Usually/always wears sunscreen:** (I: 77/200 = 38.5%) vs (C: 64/158 = 40.5%) - **Usually/always seeks shade:** (I: 76/200 = 38%) vs (C: 60/158 = 37.8%) - **Usually/always wears deliberately skimpy clothing:** (I: 45/200 = 22.5%) vs (C: 37/158 = 23.41%) | VicHealth Discovery Grant, The Australian Government, Monash University Faculty of Medicine; NHMRC |
| Armstrong, 2009 (United States) | Parallel RCT | The general population in the Boston area  Age:  ≥18 years of age  The mean age was 33.6 (range 18 – 72)  Inclusion: own a cell phone with text-message features, and demonstrate the ability to retrieve text messages. | I: 35; C: 35 | **Intervention**  The intervention group received text messages reminders daily during mornings in order to remind users to apply sunscreen. Investigators developed an electronic monitor for topical agents (sunscreen) that was adaptable to tubes for intervention participants. Each time the cap on the tube was removed, the monitor sent a message to a central repository in order to register dates and times of openings.   1. **What:** Messages consisted of 2 components: a text detailing daily local weather information and a text reminding users to apply sunscreen. 2. **When and how much**  - Date: messages were delivered during the autumn of 2007. - Frequency: daily - Hour: between 6:30 a.m. and 7 a.m. - Duration: six weeks  1. **Tailoring:** Interventions were not tailored. 2. **How well (planned):** Adherence or fidelity were not assessed.   **Control** The control group did not receive reminders. Investigators developed an electronic monitor for topical agents (sunscreen) that was adaptable to tubes for control participants. Each time the cap on the tube was removed, the monitor sent a message to a central repository in order to register dates and times of openings. | Interventions were not tailored. | **Adherence to sunscreen application**: number of days that the participant applied sunscreen divided by the total number of days of the study.  **Daily adherence rate** | 6 weeks | Adherence rate of sunscreen application: **MD: 11 (6.5 to 15.5)** | Information Systems Council of Massachusetts General Hospital and Brigham and Women’s Hospital |

I: intervention arm, C: control arm, FU: follow-up, HBM: health belief model. SSE: skin self-examination.

^1^***First* comparison*:*** (intervention: SMS + mailed sunscreen + education) vs (control: mailed sunscreen + education). ***Second* comparison*:*** (intervention: SMS + education) vs (control: education only).
