# Supplementary material for: Text message reminders for improving sun protection habits: A systematic review
Source: PLoS One. 2020 May 19;15(5):e0233220. doi: 10.1371/journal.pone.0233220 (PMC7236986; doi:10.1371/journal.pone.0233220)
Supplement: S2 Table — (DOCX) [file pone.0233220.s006.docx]

## S2 Table. Studies that were evaluated in full-text, and were excluded

| **N** | **First author** | **Year** | **Title** | **Reason for exclusion** |
| --- | --- | --- | --- | --- |
| 1 | Cheng | 2018 | Effect of stimuli on sun protective habits: A randomized double-blind controlled study | No control group. |
| 2 | Cheng | 2018 | Appearance-based vs health-based sun protective messages: A randomized, double-blind controlled study | No control group. |
| 3 | Sontag | 2017 | Assessing the Potential Effectiveness of Pictorial Messages to Deter Young Women from Indoor Tanning: An Experimental Study | Does not assess outcomes of interest. |
| 4 | Mays | 2017 | The Effects of Gain-, Loss-, and Balanced-Framed Messages for Preventing Indoor Tanning among Young Adult Women | Does not assess outcomes of interest. |
| 5 | Andersen | 2017 | A Randomized Trial of an Advanced Sun Safety Intervention for Vacationers at 41 North American Resorts | Does not use SMS text reminders. |
| 6 | Ho | 2016 | Effectiveness of a multicomponent sun protection program for young children: a randomized clinical trial | Outcomes were no assessed on the participants, but in their children |
| 7 | Evans | 2016 | Design and Feasibility of a Text Messaging Intervention to Prevent  Indoor Tanning Among Young Adult Women: A Pilot Study | Does not assess outcomes of interest. |
| 8 | Robinson | 2016 | Helping children be safe outdoors with sun protection | Full-text article was not found. |
| 9 | Robinson | 2016 | A Randomized Controlled Trial of a Mobile Medical App for Kidney Transplant Recipients: Effect on Use of Sun Protection | Does not use SMS text reminders. |
| 10 | Mays | 2016 | The influence of framed messages and self-affirmation on indoor tanning behavioral intentions in 18- to 30-year-old women | Does not assess outcomes of interest. |
| 11 | King | 2016 | Visual Exemplification and Skin Cancer: The Utility of Exemplars in Promoting Skin Self-Exams and Atypical Nevi Identification | Does not use SMS text reminders. |
| 12 | Baker | 2016 | Mediation of improvements in sun protective and skin self-examination behaviours: results from the healthy text study | Does not assess outcomes of interest. |
| 13 | Walkosz | 2015 | The Sustainability of an Occupational Skin Cancer Prevention Program | Does not use SMS text reminders. |
| 14 | Mays | 2015 | Framing Indoor Tanning Warning Messages to Reduce Skin Cancer Risks Among Young Women: Implications for Research and Policy | Does not assess outcomes of interest. |
| 15 | Finch | 2015 | User preferences for text message-delivered skin cancer prevention and early detection | Does not assess outcomes of interest. |
| 16 | Morris | 2014 | Improving the effectiveness of appearance-based sun exposure interventions with the terror management health model | Does not use SMS text reminders. |
| 17 | Hingle | 2014 | Effects of a short messaging service-based skin cancer prevention campaign in adolescents | No control group |
| 18 | Aneja | 2012 | Improvement in Patient Performance of Skin Self-examinations After Intervention With Interactive Education and Telecommunication Reminders: A Randomized Controlled Study | Intervention groups several broadcast media. It is not classified in each media. |
| 19 | Andersen | 2012 | Expanding occupational sun safety to an outdoor recreation industry: a translational study of the Go Sun Smart program | Does not use SMS text reminders. |
| 20 | Pagoto | 2010 | The sunless study: a beach randomized trial of a skin cancer prevention intervention promoting sunless tanning | Does not use SMS text reminders. |
| 21 | Lemal | 2010 | Testing the effectiveness of a skin cancer narrative in promoting positive health behavior: a pilot study | Does not use SMS text reminders. |
| 22 | Glanz | 2010 | A randomized trial of tailored skin cancer prevention messages for adults: Project SCAPE | Does not use SMS text reminders. |
| 23 | Walkosz | 2007 | Randomized trial on sun safety education at ski and snowboard schools in western North America | Does not use SMS text reminders. |
| 24 | Mayer | 2007 | Promoting sun safety among US Postal Service letter carriers: impact of a 2-year intervention | Does not use SMS text reminders. |
| 25 | Girardi | 2006 | Superiority of a cognitive education with photographs over ABCD criteria in the education of the general population to the early detection of melanoma: a randomized study | Does not use SMS text reminders. |
| 26 | Greene | 2003 | Messages influencing college women's tanning bed use: statistical versus narrative evidence format and a self-assessment to increase perceived susceptibility | Does not use SMS text reminders. |
| 27 | Mermelstein | 1999 | When tailored feedback backfires: a skin cancer prevention intervention for adolescents | Full-text article was not found. |
| 28 | Detweiler | 1999 | Message framing and sunscreen use: gain-framed messages motivate beach-goers | Does not use SMS text reminders. |
