# Supplementary material for: Text message reminders for improving sun protection habits: A systematic review
Source: PLoS One. 2020 May 19;15(5):e0233220. doi: 10.1371/journal.pone.0233220 (PMC7236986; doi:10.1371/journal.pone.0233220)
Supplement: Attachment — Submitted filename: respuesta.docx [file pone.0233220.s010.docx]

Dear editor,

We appreciate very much the comments raised by you and the reviewers. Next, we will answer to each one:

**Additional Editor Comments**

**E1C1: Please update PROSPERO – it still says only preliminary searches have been conducted.**

We made the request to update to PROSPERO website when it was required, however, we still have no answer. Regretfully, PROSPERO is presenting delays in their responses and this is out of our hands.

**E1C2: Please remove HBM acronym throughout the article. This acronym is unnecessary**

HBM acronym is not present in our paper. We replaced it for “health belief model”.

**E1C3: Change ..” lesser than ten” to “less than ten”**

Thank you for the correction. We are now using the term “less than ten”.

**E1C4: Please include reference numbers for each of the studies in tables 1-3.**

We included the reference number in each table.

**E1C5: Limitations:**

**Incorrect grammar: “A possible limitation of this review is that search was performed in only three databases, which could cause that not all published studies were found.”**

Thank you for the correction. We are now using the following sentence: *Due to the fact that search was only performed in three databases, we might not found all published studies.*

**Typo: “...such es the follow-up period”**

Thank you for the correction. We are now using the term “such as”.

**E1C6: We recommend asking a native English speaker to proofread the article.
R2C1: Final proofreading of the text should be done by a native speaker who can correct language errors.**

Before sending this manuscript for submission to PLoS One, a native English speaker proofread it.
