# Supplementary material for: Text message reminders for improving sun protection habits: A systematic review
Source: PLoS One. 2020 May 19;15(5):e0233220. doi: 10.1371/journal.pone.0233220 (PMC7236986; doi:10.1371/journal.pone.0233220)
Supplement: Attachment — Submitted filename: respuesta 4.docx [file pone.0233220.s011.docx]

Dear editor,

We appreciate very much the comments raised by you and the reviewers. Next, we will answer to each one:

**Additional Editor Comments**

**1) Please include a separate legend for each figure in your manuscript.**

We have added the legends of each figure, afther the bibliographic references, as follows:

Figure 1. Flowchart of study selection

Figure 2. Risk of bias of the included studies

Figure 3. Forest plot for the effect of SMS messages in having a sunburn anytime during follow-up
